# Atypical behavior of a black fly species connects cavity-nesting birds with generalist blood parasites in an arid area of Spain

**DOI:** 10.1186/s13071-021-04798-z

**Published:** 2021-06-03

**Authors:** Nayden Chakarov, Jesús Veiga, Ignacio Ruiz-Arrondo, Francisco Valera

**Affiliations:** 1 Department of Animal Behaviour, Bielefeld University, Bielefeld, Germany; 2 Departamento de Ecología Funcional y Evolutiva, Estación Experimental de Zonas Áridas (EEZA-CSIC), Almería, Spain; 3 Centre for Rickettsiosis and Arthropod-Borne Diseases, Hospital Universitario San Pedro-CIBIR, Logroño, Spain

**Keywords:** Ornithophilic Simuliidae, *Leucocytozoon*, Endophagous, Host specificity, Habitat choice, Nest box

## Abstract

**Background:**

The feeding behavior of bloodsucking insects determines the transmission, distribution, host spectrum and evolution of blood parasites in the wild. Conventional wisdom suggests that some vector groups (e.g. black flies, family Simuliidae) are consistently exophagous daytime biters. We aimed to understand more about the exceptions to this pattern by combining targeted trapping and molecular identification of parasites in vectors.

**Methods:**

In this study, we collected black flies in nest boxes used by European rollers *Coracias garrulus* in southeastern Spain. We molecularly analyzed 434 individual insects, identifying the black fly species caught in the nest boxes, their potential vertebrate blood meals, and the haemosporidian parasite lineages that they carried.

**Results:**

Only one black fly species, *Simulium rubzovianum*, appeared to enter the nest boxes of rollers. Among the trapped specimens, 15% contained vertebrate DNA, which always belonged to rollers, even though only half of those specimens were visibly engorged. Furthermore, 15% of all black flies contained *Leucocytozoon* lineages, indicating previous feeding on avian hosts but probably not on infected adult rollers. The known vertebrate hosts of the recorded *Leucocytozoon* lineages suggested that large and/or abundant birds are their hosts. Particularly represented were cavity-nesting species breeding in the vicinity, such as pigeons, corvids and owls. Open-nesting species such as thrushes and birds of prey were also represented.

**Conclusions:**

Our data strongly suggest that *S. rubzovianum* bites uninfected roller nestlings and infected individuals of other species, potentially incubating adults, inside nest boxes and natural cavities. This simuliid does not appear to have a strong preference for specific host clades. Contrary to the general pattern for the group, and possibly enhanced by the harsh environmental conditions in the study area, this black fly appeared to intensively use and may even have a preference for confined spaces such as cavities for feeding and resting. Preferences of vectors for atypical microhabitat niches where hosts are less mobile may enable social and within-family transmission and parasite speciation in the long term. At the same time, a lack of host preference in concentrated multispecies communities can lead to host switches. Both processes may be underappreciated driving forces in the evolution of avian blood parasites.

**Graphical abstract:**

**Figure:**
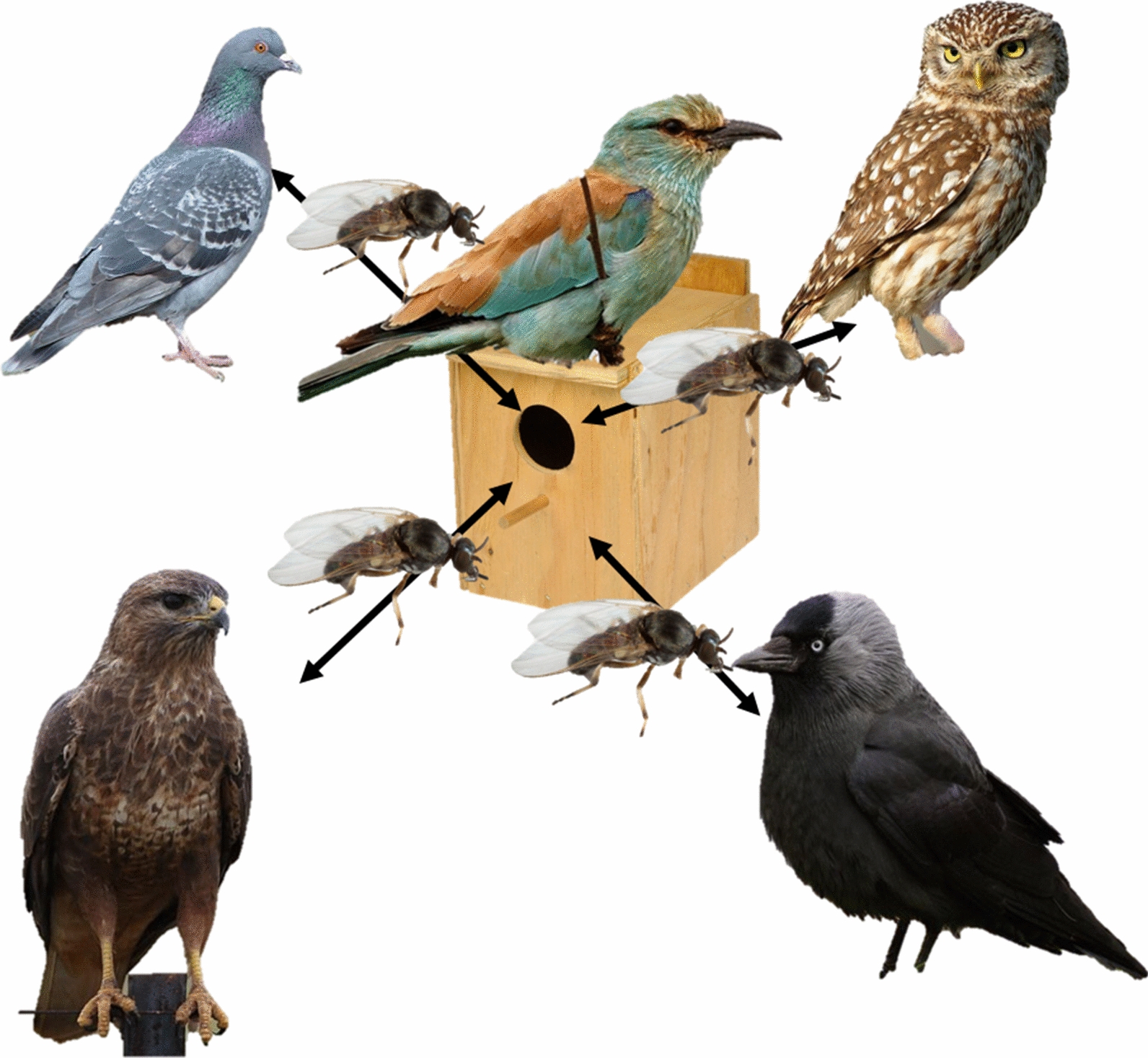

## Background

Vector-borne parasites exert major selection forces on their hosts, but the distribution of the former crucially depends on the behavior and habitat choice of the transmitting organisms [1]. Understanding the feeding behavior of potential vectors can be informative both about short-term processes relevant to health and conservation, such as increased virulence in weakened and atypical hosts, and about long-term coevolutionary processes [2–5]. However, studies of the natural foraging behavior of insect vectors in the field are often constrained by their size and mobility. Thus, parasites present in vectors can be used as handy markers of host-parasite interactions, as they can inform simultaneously about the current members of exchange networks and the potential for rare evolutionary events such as host shifts [6].

Black flies (Diptera, Simuliidae) are one of the main groups of bloodsucking dipteran vectors, with members of the family transmitting haemosporidian parasites, trypanosomes and filarial nematodes [7]. Compared to other bloodsucking dipterans transmitting human diseases, black flies have been less investigated and may be harder to study. The feeding ecology of black flies is not well understood because colonies of very few species have been established in laboratories and feeding behavior is almost never performed in captivity. Adult black flies are commonly trapped using CO_2_-baited traps [8–12]. Valuable information complementary to that obtained with classic methods can be gathered by investigating the wide spectrum of habitat niches and hosts that attract different black fly species. Alternative approaches to black fly trapping, such as net-scooping and use of living hosts as bait, have been used [8, 9, 13–15]. Black flies seem to prefer either common and/or large hosts [14, 16]. Black fly species are distinguishable as either mammalophilic or ornithophilic, but blood meals of engorged black flies in Scandinavia suggest species-specific preferences for different bird host groups [17]. At the same time, the host species composition and parasite loads of black flies in central Europe suggest lack of host specificity for some ornithophilic black fly species but a preference for specific habitat niches, e.g. the upper forest canopy [14, 18]. Correspondingly, habitat and nest height explain the prevalence of black fly-transmitted *Leucocytozoon* lineages observed in host bird assemblages, while cavity nesting increases the probability of high *Leucocytozoon* prevalence in bird hosts [19].

It is important to note that bird hosts live in complex environments that offer many microhabitat niches. Habitat preferences of vectors can lead them to have more frequent contact with certain host species, e.g. open-nesting birds breeding high in the canopy such as raptors, corvids and thrushes, or cavity-nesting birds such as diverse passerines and owls [14, 20]. Thus, the specificities of black fly behavior may lead to separate parasite-exchange networks between species sharing similar niches within a given habitat. Vectors can potentially enhance parasitic host-switching rates by increasing the incidence of spillover between niche-sharing host species. This process may select for adaptability of parasites to novel host groups and blood environments, and eventually also lead to the establishment of new predominant host-parasite relationships and a consequent increase in co-speciation rates [21, 22]. However, the differences in vector behavior and the dominating vector species in most microhabitats are still largely unknown even in the best studied areas of the world.

Ornithophilic simuliids are the essential vectors of avian haemosporidian parasites of the genus *Leucocytozoon* [22]. Members of this genus have been recently shown to have the higherst rates of co-speciation and host switching among blood parasites, but the vector behaviors which may lead to these patterns remain to be revealed [23]. A great diversity of genetic *Leucocytozoon* lineages has been described and systematized in recent years, along with the vertebrate hosts in which they occur [12, 24]. This allows *Leucocytozoon* lineages to be used as natural markers for the feeding preferences and foraging habitats of black flies in which they are molecularly detected [6, 14].

In this study, we aimed to explore the feeding behavior of black flies to assess whether they are really mainly exophagous feeders. For this, we studied the system formed by European rollers, *Coracias garrulus* (hereafter ‘rollers’) breeding in nest boxes, and the black flies visiting them. We also studied which *Leucocytozoon* lineage composition they carry. Many black fly species suck blood from hosts of different taxonomic orders. Therefore, our hypothesis was that cavity-feeding black flies will bear parasites of most host species breeding locally in nest boxes and possibly also in the nest box surroundings. Since microhabitats such as cavities and burrows may demand particular behavioral or morphological adaptations, and since black flies are generally outdoors (exophilic) biters, we predicted that only few species might forage on cavity-nesting birds [7]. The samples were collected in an arid area in southern Spain, where the breeding habitat for black fly larvae—running water—is scarce and frequently brackish. Harsh environmental conditions can make nest boxes an attractive, relatively humid microhabitat, and thus they could become an important reservoir of black fly diversity [25]. In contrast to most other local birds, rollers are exclusive cavity breeders and are the most common cavity breeders in the study area. Black flies were collected in the framework of a long-term project on host-parasite interactions of cavity-nesting birds.

## Methods

### Study area

The study area and specimen collection methods largely correspond to those of a previous study [26]. In brief, the samples were collected in a 50-km^2^ area in the Tabernas Desert (Almería, southeastern Spain; 37°05′N, 2°21′W). The landscape mostly consists of patches of shrubby vegetation and olive and almond groves interspersed among dry watercourses and ravines. Inhabited farms are scarce and scattered along the study area. The climate is temperate, semiarid Mediterranean with a strong water deficit during the long, hot summer months (June–September), when the absolute maximum monthly temperature is higher than 40 °C and the monthly average maximum daily temperature remains above 30 °C. The average annual temperature is 18 °C, with mild inter-annual oscillations of 3–4 °C and significant intra-annual fluctuations. The mean annual rainfall is ca. 230 mm with high inter-annual and intra-annual variability [27].

The bird community can be divided into open-nesting species (small passerines, and some species of doves and pigeons) and cavity-nesting ones. The latter breed mainly in natural cavities in dry ravines (i.e.* ramblas*, temporary stream channels with steep sandstone banks) and human-made constructions [28]. Among the cavity-nesting species in the study area are the common kestrel (*Falco tinnunculus*), jackdaw (*Corvus monedula*), Eurasian hoopoe (*Upupa epops*), rock pigeon (*Columba livia*), little owl (*Athene noctua*) and Eurasian scops owl (*Otus scops*). The European roller and rock pigeon are, by far, the most abundant ones. As a result of a long-term nest box scheme which started in 2005, most rollers breed in wooden nest boxes (height × length × width, 310 × 232 × 230 mm; entrance diameter, 60 mm; with a removable upper lid to allow nest monitoring) installed on eucalyptus trees, sandstone banks and isolated and deserted country houses [29, 30]. Rollers are migratory birds wintering in Africa and arriving at the breeding grounds in the study area at the end of April, when resident, secondary cavity-nesting birds are already settled. Eggs (mean clutch size = 4.23) are incubated by both sexes for ca. 21 days [31] and hatching is distinctly asynchronous. Rollers rear a single brood per year with fledglings leaving the nest approximately 22–25 days after hatching in the studied population.

### *Simulium* trapping

*Simulium* spp. specimens were trapped using sticky traps placed in nest boxes (*n* = 36) occupied by rollers between 6 June and 23 July 2018, i.e. during the nestling stage when roller chicks were between 13–14 and 21–22 days old. We followed the method described by Tomás et al. [32] (i.e. using Petri dishes smeared with body gel oil as a non-attractant glue) but replaced the Petri dishes with white vegetal paper (175.5 cm^2^) that was fixed by thumbtacks on the inner side of the upper lid. The sticky paper was replaced every 4 days to avoid desiccation; two traps were placed per nest and trapping occurred for 8 consecutive days. Additionally, *Simulium* were opportunistically caught at the nests by hand during routine visits, and all specimens were preserved in 95% ethanol and frozen until identification. The thorax and the abdomen of engorged black flies were separated and analyzed independently.

### Morphological identification of black flies

From the total black flies captured during 2018 a subsample of 68 were used for the combined analysis of morphological and molecular identification. Individuals were identified based on descriptions given in the keys of Belqat et al. [33] and Rivosecchi et al. [34]. Morphological identification was facilitated by examining preparations of specimen genitalia mounted on slides.

### Molecular identification of black flies, host blood and haemoparasites

Sample and data analyses were performed as described previously [14]. Briefly, DNA of single black flies was extracted from complete specimens or separated abdomen and thorax (in engorged black flies) using overnight digestion in 490 µl of QIAGEN ATL lysis buffer and 10 µl of Proteinase K followed by a standard phenol–chloroform protocol. DNA was quantified using a NanoDrop spectrophotometer (Thermo Fisher Scientific, Waltham, MA). A modified Hotshot technique was used for the DNA extraction of each individual of the subsample for morphological identification [35]. Samples were screened with three separate polymerase chain reaction (PCR) assays: (i) black fly species was determined for a subsample of 126 individuals with conserved primers targeting the cytochrome *c* oxidase subunit 1 DNA region (LCO1490, 5′-GGT CAA CAA ATC ATA AAG ATA TTG G-3′; and HCO2198, 5′-TAA ACT TCA GGG TGA CCA AAA AAT CA-3′) [36]; (ii) presence of vertebrate host DNA was tested for a subsample of 176 individuals with conserved primers targeting the vertebrate cytochrome *b* (L14841, 5′-AAA AAG CTT CCA TCC AAC ATC TCA GCA TGA TGA AA-3′; and H15149, 5′-AAA CTG CAG CCC CTC AGA ATG ATA TTT GTC CTC A-3′) [37]; and (iii) presence of haemosporidian lineages within the black fly individuals was established for a subsample of 434 individuals with a nested PCR following Pérez-Rodríguez et al. [38], using the primer pair Plas1 (5′-GAG AAT TAT GGA GTG GAT GGT G-3′) and HaemNR3 (5′-ATA GAA AGA TAA GAA ATA CCA TTC-3′) for the first PCR and the internal primers 3760F (5′-GAG TGG ATG GTG TTT TAG AT-3′) and HaemJR4 (5′-GAA ATA CCA TTC TGG AAC AAT ATG-3′) for the second PCR. This nested PCR primer protocol amplifies the cytochrome *b* gene of all haemosporidian genera, including *Leucocytozoon* lineages that are less well detected with other nested PCR protocols, e.g. *Leucocytozoon* of raptors [24; personal observation]. A *Leucocytozoon*-infected sample from an adult roller was additionally amplified to ensure that the used nested PCR protocol can detect roller-infecting haemosporidian lineages. PCR products were run on 2% agarose gels. Amplicons were purified with ExoSAP (Thermo Fisher Scientific) and bidirectionally sequenced on an ABI 3730 Analyzer (Applied Biosystems, Waltham, MA) with the BigDye Terminator v1.1 cycle sequencing kit (Thermo Fisher Scientific) using the respective two primers. Raw sequences were edited and aligned in Geneious 8.1.9 (www.geneious.com) and compared with sequences on GenBank or, in the case of 3760F/HaemJR4, with sequences on the MalAvi database, as of 28 July 2020 [24].

## Results

A total of 515 black flies (range 0–199 per nest) were collected by sticky traps in nest boxes occupied by rollers during 2018. Black flies were captured in 29 out of 36 roller nest boxes (prevalence = 0.81; confidence interval 95% = 0.64–0.92; mean intensity ± SE = 17.8 ± 6.95). Of these, 13 individuals were visibly engorged. Two additional black flies were captured manually in one roller and one scops owl nest box.

### Morphological identification

Morphological identification of the subset of 68 black fly individuals revealed the presence of a single morphospecies: *Simulium rubzovianum.* All morphologically inspected individuals caught in nest boxes were females.

### Molecular identification of black flies, host blood and haemoparasites

Black flies species barcoding of the subset of 68 individuals morphologically identified and a subset of 126 individuals identified only by molecular barcoding revealed that all of them belonged to the species *S. rubzovianum*. Barcoding of all individuals clustered together in the neighbor joining tree (not shown).

Barcoding of 176 black flies for vertebrate DNA showed that 26 specimens contained DNA of rollers, *C. garrulus*, and one further sample of a manually caught black fly in a scops owl nest box contained DNA of this bird species. Only 12 of the 26 specimens with roller DNA were visibly engorged. In two engorged black flies, vertebrate DNA could be identified both from the thorax and abdomen, in 11 cases (including one black fly with scops owl DNA) only from the abdomen, and in two cases of apparently engorged individuals no vertebrate DNA could be amplified.

**Fig. 1:**
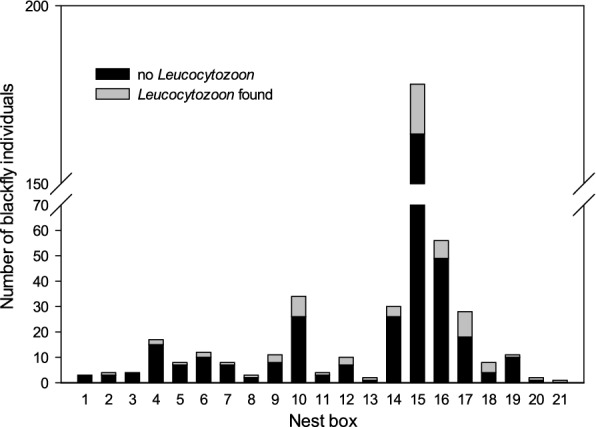
Number of *Simulium rubzovianum* caught at separate nest boxes inhabited by Eurasian rollers *Coracias garrulus* including number of specimens in which *Leucocytozoon* was detected (*grey*, *n* = 66). The figure is based on a subset of 434 black fly individuals caught in 21 nests

Haemosporidian barcoding of a subset of 434 black flies from 21 nests revealed that 63 individuals contained one *Leucocytozoon* lineage while three others contained two lineages of *Leucocytozoon* which could be identified (Fig. 1; Table 1). This corresponded to 15.2% (confidence interval 95% = 11.96–18.94%) overall *Leucocytozoon* prevalence in the collected black flies.

**Table 1:** Haemosporidian lineages found in black flies from nest boxes of European rollers in southeastern Spain

Lineage found in *Simulium rubzovianum*	Black flies infected by lineage (*n* = 434)	Typical hosts^a^ and % similarity with closest lineage
AEMO02	9	*Aegypius monachus, Columba livia, Columba palumbus, Streptopelia turtur*
ATNO1	2	*Athene noctua*
COCOR12	4	*Corvus corone, Columba livia*
COLIV04	13	*Columba palumbus, Streptopelia turtur*
COLPAL04	2	*Columba palumbus*
CYACOO01	1	*Cyanopica cooki*
CYACOO03	1	*Cyanopica cooki*
EMCIR02	1	*Fringilla coelebs, Emberiza cirlus, Sylvia melanocephala*
GAGLA06	6	*Corvus corax, Garrulus glandarius*
HAWF7	1	*Coccothraustes coccothraustes, Luscinia cyanura*
MILANS04	5	*Buteo buteo, Accipiter gentilis, Milvus migrans, Milvus milvus*
MTUR2	1	*Turdus viscivorus*
NEVE01	4	*Turdus merula*
OTSCO01	1	*Otus scops*
OTSCO09	1	*Otus scops*
PAHIS03	1	*Passer hispaniolensis*
PARUS69	1	*Certhia brachydactyla, Cyanistes caeruleus, Fringilla coelebs, Sylvia melanocephala*
PARUS90	1	*Cyanistes caeruleus, Parus major*
STRORI02	1	*Streptopelia turtur*
STAL1	1	*Strix aluco*
TUMER01	6	*Passer domesticus, Turdus merula*
SIMRUB1^a^	3	Corvidae, 97.5% to PICPIC01 of *Corvus corax, Corvus monedula, Parus major, Pica pica*
SIMRUB2^a^	1	Turdidae, 95.4% to TFUS15 of *Catharus fuscater*
SIMRUB3^a^	1	Turdidae, 99.8% to TUMER18 of *Turdus merula*
SIMRUB4^a^	1	Accipitriformes, 92.6% to BUTJAM04 of *Buteo jamaicensis*
Total infected black flies		66
Total lineage occurrences		69

All black fly specimens belong to *S. rubzovianum*. All the parasite lineages belong to the genus *Leucocytozoon* and are listed with their previously reported avian host species*.* Three vector individuals were co-infected by two lineages (see details in the main text), which are listed as independent occurrences

^a^Previously undescribed lineages (SIMRUB1-4) are indicated with known avian hosts of closest BLAST matches

Only in two instances were both parasite and avian DNA present in black flies—in one case scops owl DNA was found together with DNA of the *Leucocytozoon* lineage OTSCO01. In another specimen, roller DNA was present along with DNA of lineage COLIV04.

The known host groups for the identified *Leucocytozoon* lineages were doves and pigeons (28 cases, including one co-infection), corvids (28 cases), diurnal raptors (15 cases), thrushes (13 cases, including one co-infection), sparrows (7 cases), owls (4 cases, including one co-infection), finches (3 cases) and tits and warblers (2 cases each). In all three cases of mixed infections, the co-infecting lineages appeared to be typical of the same host group—STAL1 and OTSCO09 are typical of owls, AEMO02 and COLPAL04 are typical of pigeons [39], and TUMER01 and NEVE01 are thrush parasites [40]. Three of the most represented *Leucocytozoon* lineages (AEMO02, COCOR12, COLIV04), comprising 26 of the 69 lineage occurrences, are generalist *Leucocytozoon* parasites which have been shown to infect host species from different avian orders (Table 1).

## Discussion

To our knowledge, this is one of the first studies of a local black fly fauna that utilizes the specific niche constituted by nest boxes, which potentially corresponds to the use of natural tree and rock cavities [7, 20, 41]. Additionally, the data on the *Leucocytozoon* lineages found in these potential vectors in southeastern Spain indicate their natural host spectrum, feeding habitat range and preferences.

### Cavity use and feeding habits of black flies

Based on morphological and molecular identification, *S. rubzovianum* was the only species of black fly found in this study in the nest boxes of European rollers in southern Spain.

The general rule is that simuliids are daytime exophagous, i.e. outdoors biters. Most species are reluctant to feed even in large enclosed rooms, making them particularly hard to maintain in laboratory colonies [7]. Although some exceptions have been reported that suggest that feeding by simuliids on small mammals in burrows or on cavity-nesting birds does occur [41–43], endophagous feeding of simuliids in dark, confined and narrow spaces has not been documented. After feeding, simuliids have decreased mobility and often rest in places where they are unlikely to be found, e.g. tree crowns, or confined spaces such as within leaf litter, on nest branches, in crevices and potentially burrows [7, 14]. This impedes making a distinction between “feeding and resting in the nest box” and “feeding around the nest box and resting inside”.

However, several of our observations may help us to clarify whether the black flies were actually feeding in the nest boxes: (i) all morphologically inspected individuals caught in the nest boxes were females, which suggests that black flies enter nest boxes specifically to feed and not solely to rest or seek shelter, as the latter would equally apply to males; (ii) black flies trapped in nest boxes occupied by rollers had *Leucocytozoon* lineages specific to other co-existing bird species. However, in no case did we find black flies that contained blood of these species, which suggests that these flies had previously fed, and after completing the gonotrophic cycle, entered the nest boxes for their next meal; (iii) all 12 freshly engorged specimens trapped in nest boxes with roller nestlings had roller blood. European rollers are standard hosts of two morphospecies of *Leucocytozoon—Leucocytozoon eurystomi* and *Leucocytozoon bennetti*—with the prevalence of the latter exceeding 50% in some avian populations [6]. Previous studies based on smear screening found ca. 30% prevalence of *Leucocytozoon* in adults of a focal roller population [44]. This was probably an underestimate of the infected adults since microscopic examination can have low sensitivity [45]. In fact, the prevalence of *Leucocytozoon* in adult rollers from the study area estimated by molecular techniques is ca. 90% (Veiga et al., in preparation). Therefore, at least some blood meals originating from adult breeding rollers should carry detectable *Leucocytozoon* DNA. However, only in one case did the engorged black fly also contain *Leucocytozoon* DNA, and even in this case the lineage was typical of pigeons and corvids. In the other cases, the blood meal could have come from uninfected adults or, more probably, from nestlings, since blood of young nestlings is much less likely to contain parasite DNA*.* Revisiting the same feeding sites and even boxes by black flies and other vectors may commonly lead to quasi-vertical transmission of parasites—from adults to offspring via the vector—thus creating a prevalence structure based on host families [46]. Support for such patterns of infection has been found in other hosts of *Leucocytozoon* [47, 48]. However, engorged black fly vectors would need two sufficiently long time windows to bite and transmit parasites previously ingested from incubating adults: (i) several days or weeks to lay eggs at a water body and develop infective sporozoites in the salivary glands; and (ii) a window when nestlings have not fledged and can be bitten. This would be followed by a prepatent period before the development of blood stages in nestlings, which typically takes more than 10 days for *Leucocytozoon* [6]. This makes the presence of *Leucocytozoon* blood stages in 2- to 3-week-old nestlings less probable.

All these arguments suggest that nestlings are the likely donors of uninfected roller blood meals and that the sampled *S. rubzovianum* individuals had indeed foraged in the confined darkness of the nest box. It should be noted that under the scenario of family-based social transmission, the closest interactions are between parents and offspring and thus most transmission events may occur before young fledge. This would select for short prepatent periods, followed by a peak in infection intensity allowing for some extra transmission between siblings. Such a short prepatent period may account for the black fly that contained both roller and parasite DNA, which was collected in the nest box with the oldest nestlings of the study, which were very close to their fledging age. However, since the lineage found has been cited for pigeons and corvids, it is more likely that this black fly previously fed on pigeons and then on an uninfected roller. A further specimen engorged with infected scops owl blood (in this case with a *Leucocytozoon* lineage found in Strigiformes) was caught in a nest box during a regular incubation check, also suggesting that it fed in the box shortly before it was caught. Specific studies on the development of parasitemia over the lifetime of hosts and on the lineages of *Leucocytozoon* found in rollers are needed to highlight the potential vectorial role of black flies in our roller population.

While it seems very likely that *S. rubzovianum* feeds in nest boxes and even in natural cavities, this type of behavior does not necessarily apply to all ornithophilic species. Previous studies found *Simulium* (*Eusimulium*) *petricolum* to be another ornithophilic simuliid species present in this type of habitat and entering nest boxes [44]; however, we could not find any evidence that *S. petricolum* used nest boxes as foraging or resting sites in 2018. Other black fly species reported using nest boxes, in the Czech Republic, are *Simulium* (*Nevermannia*) *vernum* and *Simulium* (*Eusimulium*) *angustipes* [20]. This study [20] confirmed *Eusimulium* and *Nevermannia* as the two main ornithophilic subgenera of black flies in Europe and further suggested that more species of *Eusimulium* are prone to exploit cavities as a microhabitat niche. At this point, we cannot conclude whether *S. rubzovianum* is the only, or the most frequent, black fly species present in the dry river beds of our study area, or whether it is the only species attracted to nest boxes or to roller olfactory cues. To be able to make a clearer distinction between the locally available vector fauna and actual users of a given microhabitat, we would recommend simultaneous trapping inside and outside of the microhabitat. With regard to simuliids, this calls for less established and unorthodox methods of catching vectors such as sticky traps or scoop netting [7, 14, 17]. Nonetheless, for such trapping methods, attractants are also necessary and bird-inhabited cavities may be among the most obvious attractants, which simultaneously lead to a higher diversity of potential avian hosts in this habitat [30].

### *Leucocytozoon* lineage composition and host preference

The composition of *Leucocytozoon* lineages found in the black flies trapped in nest boxes is also informative of their host range and foraging habitat. Lineages known from doves, pigeons, corvids and owls were well represented in the present study. Some known sources of these parasites are other secondary inhabitants of cavities present in this habitat—rock pigeons *C. livia*, jackdaws *C. monedula* and little owls *A. noctua*. Indeed, roller nest boxes installed on cliffs and on farms are often very close to cavities, whether artificial or natural, used by these species [30]. However, none of the parasite-bearing black flies contained DNA of these hosts. This suggests that black flies feed on infected hosts of these species, whether in other cavities or in the open. Only after spending sufficient time for complete blood digestion, egg development, and search and return from an oviposition site, do they enter cavities again, likely in search of new hosts. For parasites of colonial host species, this may lead to less structured transmission than outlined above (e.g. quasi-vertical) and allow exchange between families of different colony members, which may belong to the same or to different host species.

It should be noted that the presence of some *Leucocytozoon* lineages in *S. rubzovianum* does not prove that this species is their competent vector. As outlined above, *Leucocytozoon* transmission between rollers in the area remains elusive. Since rollers are Afro-Palearctic migrants, transmission of their *Leucocytozoon* parasites may occur predominantly in Africa, rather than at the breeding grounds, but reasons for the latter remain to be identified. Transmission experiments show that most well-studied *Leucocytozoon* species develop in diverse black fly species [6, p. 80 and references therein]*.* Therefore, the ecology of black fly vectors may be a greater limitation for the transmission of different *Leucocytozoon* species than incompatibility with the vector. Factors such as vector species-specific interactions with *Wolbachia* symbionts and microhabitat preferences may strongly determine which vector individuals transmit which parasites between which hosts and where [49]. Further studies with more comprehensive sampling strategies are needed to elucidate such details of the host-vector-parasite exchange networks.

Despite a strong bias towards hole-nesting species, some of the lineages found in this study are only known from thrushes, diurnal raptors and warblers, whose representatives in southeastern Spain only breed in open nests. Additionally, black flies in this habitat have been found to be more common and abundant in nest boxes on trees than on cliffs [50]. Therefore, similarly to other black fly species, *S. rubzovianum* may be attracted to wooded areas and feed both on open- and cavity-nesting species, thus utilizing the full range of microhabitats where avian hosts can be easily attacked. The propensity to feed naturally in closed spaces makes *S. rubzovianum* a good candidate for breeding in laboratory conditions, if its broad habitat tolerance also extends to mating and ovipositing [7]. It remains open whether the apparent common use of holes by this species is due to an actual preference for this microhabitat or is driven by the harsh climatic conditions of the area. Given the overall avifauna of the habitat where rollers breed, the composition of *Leucocytozoon* lineages shows representation of host species which are either abundant or relatively large bodied, thus confirming a pattern that has been established at other locations [8, 14].

So far, no *Leucocytozoon* lineages have been molecularly described from roller samples that might allow identification of the morphospecies infecting this host group. Several of the most common lineages found in our study are known to infect pigeons as well as corvids and raptors. This very wide range of hosts across the avian phylogeny might encompass rollers and make them potential hosts of these generalist lineages. It remains to be shown if this is really the case and whether infection of distant host groups leads to differences in the morphology of *Leucocytozoon* generalist lineages. To achieve this aim, it is highly recommended to make blood smears when collecting avian blood samples for DNA analyses.

## Conclusions

Insights into the behavior and ecology of vectors are scarce although they are both indispensable and key to understanding the transmission dynamics of parasites, and their consequences for host populations and multi-host assemblages [22]. The differential use of some niches within a given habitat by some vectors may be additionally elucidated by analyzing host networks and using unconventional sampling methods. Our study utilized a habitat (i.e. nest boxes) supplementation programme, combined with microhabitat-targeted trapping and extensive knowledge of bird-haemosporidian associations, carefully curated by devoted colleagues in recent years [24]. This powerful combination of factors shows that in arid habitats a sole vector species may connect hosts of different microhabitat niches by feeding and potentially transmitting numerous lineages both inside and outside of breeding cavities. Most details about habitat and host specializations of vectors remain to be revealed and the use of parasites as biological markers can be a potent approach to achieving these aims [6].

## Data Availability

All data supporting the conclusions of this article are included in the article.
